# Supplementary material for: Propolis Alleviates Acute Lung Injury Induced by Heat-Inactivated Methicillin-Resistant Staphylococcus aureus via Regulating Inflammatory Mediators, Gut Microbiota and Serum Metabolites
Source: Nutrients. 2024 May 24;16(11):1598. doi: 10.3390/nu16111598 (PMC11175110; doi:10.3390/nu16111598)
Supplement: Supplementary file 1 [file nutrients-16-01598-s001.zip › nutrients-2997097-supplementary.pdf]

Supplementary materials

Figure S1 Statistical validation of the corresponding OPLS-DA model by permutation analysis in the negative ion model. Q2 is the predictive ability of the model.

Figure S2 Statistical validation of the corresponding OPLS-DA model by permutation analysis in the positive ion model. Q2 is the predictive ability of the model.

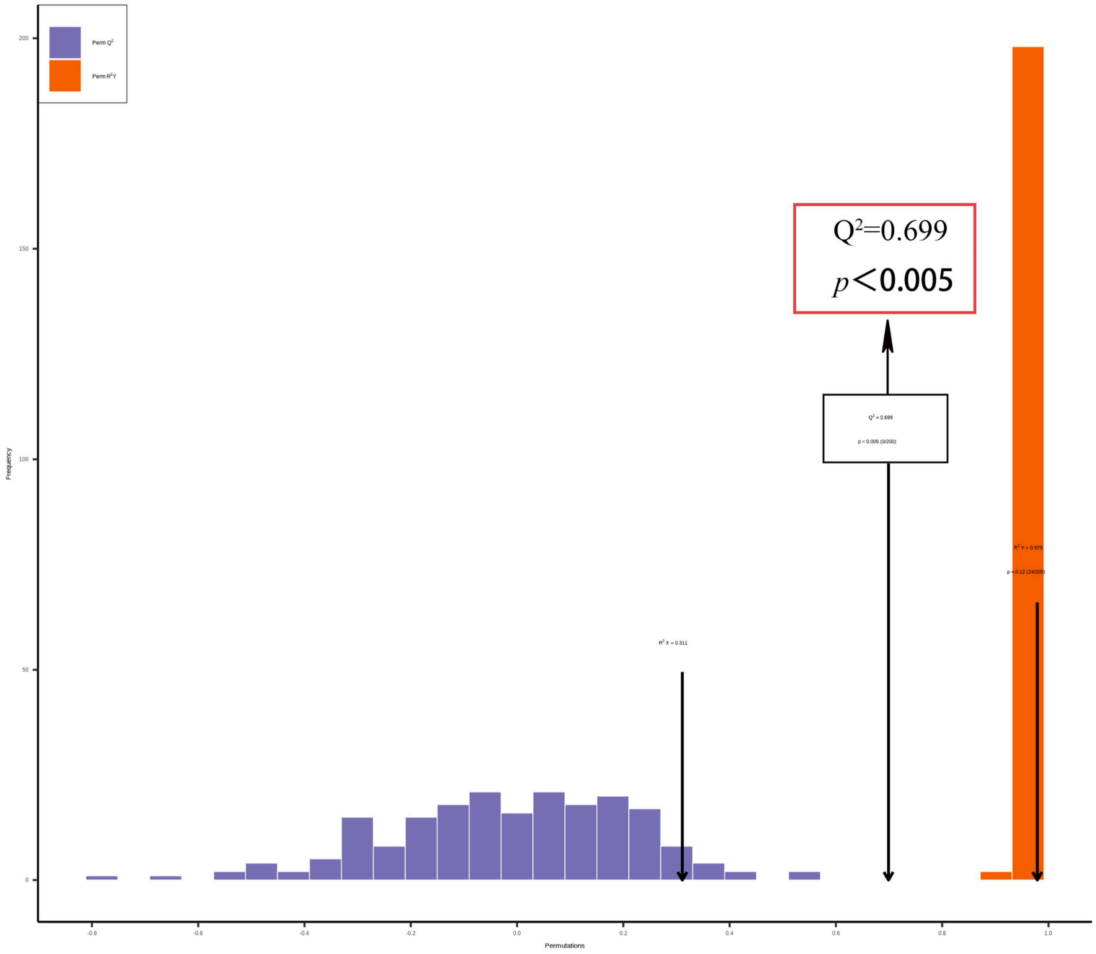

Figure S1

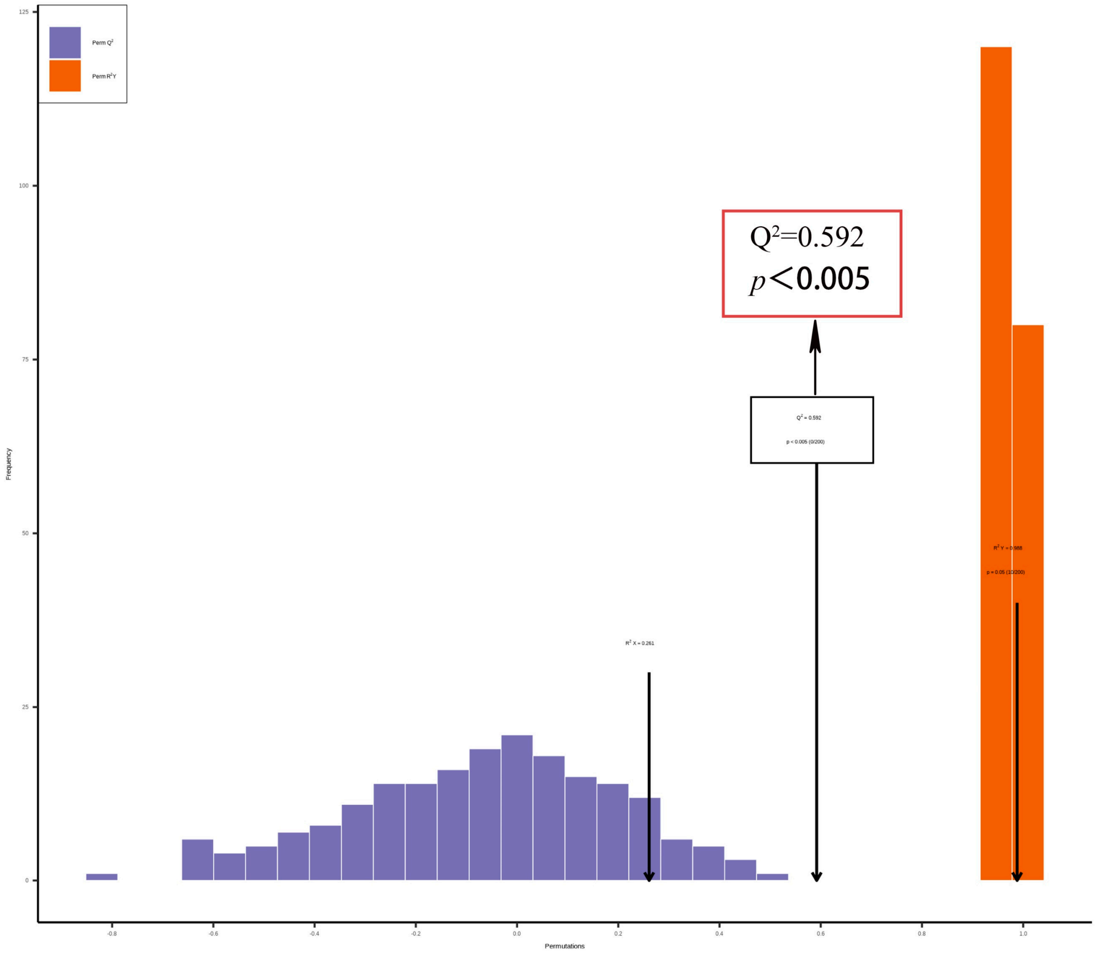
Perm Q^2
Perm R^{2}Y
Q^2=0.592
p<0.005
Q^2 = 0.592
p < 0.005 (0/200)
R^2 Y = 0.988
p = 0.05 (10/200)
R^2 X = 0.261
Frequency
Permutations
125
100
75
50
25
0
-0.8
-0.6
-0.4
-0.2
0.0
0.2
0.4
0.6
0.8
1.0

Figure S2
